# A Cost-Effective Approach for Non-Persistent Gold Nano-Architectures Production

**DOI:** 10.3390/nano10081600

**Published:** 2020-08-14

**Authors:** Giulia Giannone, Melissa Santi, Maria Laura Ermini, Domenico Cassano, Valerio Voliani

**Affiliations:** 1 Center for Nanotechnology Innovation@NEST, Istituto Italiano di Tecnologia, Piazza San Silvestro, 12-56127 Pisa, Italy; giulia.giannone@sns.it (G.G.); melissa.santi@sns.it (M.S.); laura.ermini@iit.it (M.L.E.); domenico.cassano@sns.it (D.C.); 2 NEST-Scuola Normale Superiore, Piazza San Silvestro, 12-56127 Pisa, Italy

**Keywords:** nanomaterials, theranostics, gold nanoparticles, production, radio-oncology

## Abstract

The effective exploitation of the intriguing theranostic features of noble metal nanoparticles for therapeutic applications is far from being a routine practice due to the persistence issue. In this regard, passion fruit-like nano-architectures (NAs), biodegradable and excretable all-in-one, nature-inspired platforms which jointly combine these characteristics with the appealing optical behaviors of noble metal nanoparticles, can offer a new alternative for theranostic applications. Besides the need for efficacious and innovative systems, the reliable and cost-effective production of nanomaterials is a pivotal subject for their translation to the clinical setting. Here, we demonstrate the production of a new cheaper class of degradable, ultrasmall-in-nano-architectures (dragon fruit NAs, dNAs) using polyethyleneimine (PEI) as a cationic polymer without affecting either their compositions or their physiological behaviors, compared to the previous NAs. In particular, the standardized protocol characterized in this work ensures the preparation of high gold-loading capacity nanoparticles, a peculiar characteristic that, synergically with the interesting properties of PEI, may unlock new possible applications previously precluded to the first version of NAs while reducing the hand-made production cost by three orders of magnitude.

## 1. Introduction

Notwithstanding the considerable progress that has been made in clinical settings in the last decade, several diseases, among which are various neoplasms, still result in a poor prognosis [1]. In this regard, noble metals, due to their unique optical properties, chemical stability, ease of synthesis, and functionalization, are particularly appealing for the development of enhanced or combined treatments [2,3]. Indeed, nanostructured noble metals can be exploited, for example, for their peculiar interactions with electromagnetic radiation for theranostic applications, biosensors, and drug delivery systems [4,5,6,7]. The biodegradation and excretion of nanomaterials is a pivotal topic in medicine, and the clinical translation of noble metal nanomaterials has been limited by their long-term persistence in organisms and potential toxicity [8].

In recent years, we have addressed this concern by introducing the ultrasmall-in-nano approach, which combines the theranostic features of noble metal nanoparticles with their renal excretion capability [3,9,10]. Within this approach, passion fruit-like nano-architectures (NAs), inorganic nanoplatforms composed of (bio)degradable hollow silica nanospheres enclosing a polymeric matrix made of poly(sodium 4-styrenesulfonate) (PSS), poly(L-lysine) (PL), and plasmonic ultrasmall nanoparticles (USNPs), have been produced [11].

The silica shell can be easily functionalized, offering protection to the encapsulated moieties against the environment until its degradation occurs, and can exploit the permeability and retention effect (EPR) [12].

Their (bio)degradation to metabolic-friendly products, as well as their quantitative excretion, biosafety, biokinetics, and potential applications, have already been demonstrated [3,4,9,10,13,14].

Beyond metal persistence and bio-nano interactions, the major requirements for a successful scale-up and clinical translation of metal nanoplatforms are related to the production, which should be cost-effective and reproducible [15,16]. For this purpose, the synthetic set-up should be easy and versatile, and the employed materials must be cheap and available on a large scale.

Here, we report a reproducible and standardized method for the production of a new class of cost-effective degradable nanoarchitectures (dragon fruit NAs, dNAs), replacing the poly(L-lysine) (PL) with the cationic polymer polyethyleneimine (PEI). Remarkably, PEI is a cationic-branched chain polymer characterized by a high positive charge due to the presence of several primary, secondary, and tertiary amine groups, disclosing a new panorama of potential applications due to the improved properties of this nanosystem. PEI is biocompatible and approved for several applications, among which are human medical ones [17]. Moreover, PEI can enhance the efficiency of drug delivery systems by promoting the endosomal escape of drugs because of the proton sponge effect resulting from its charged side groups [18].

The hand-made production cost of dNAs is reduced by three orders of magnitude, with respect to NAs, facilitating potential translation to patients. In this regard, we have estimated that the cost of hand-made NAs and dNAs is almost completely associated with the price of the poly(L-lysine), whose market price is about 1000 €/g, much higher than polyethyleneimine, which is less than 1 €/g [19]. Overall, the possibility to produce NAs employing PEI has further demonstrated the robustness of the NA’s production protocol and resulted in the composition of a novel nano-platform with additional intriguing features, with respect to standard NAs. Furthermore, their internalization behaviors, as well as their cytotoxicity trends, have been assessed on three different cell lines of particular interest for their potential applications, confirming their low toxicity profiles [3,9,10]. 

## 2. Materials and Methods

All chemicals were purchased from Sigma-Aldrich (St. Louis, MO, USA) unless otherwise specified. All chemicals were used as received.

### 2.1. Synthesis of Nano-Architectures

#### 2.1.1. Synthesis of Dye-Modified Polyethyleneimine

Functionalized polyethyleneimine dye is prepared by mixing 200 μL of branched polyethyleneimine (10 µL/mL) (average Mn ~1800, 50 wt.% aqueous solution), with an AlexaFluor-680-N-Hydroxysuccinimide (NHS) ester (100 μg, Invitrogen, Carlsbad, CA, USA) in phosphate-buffered saline (PBS) (200 μL). The solution was stirred overnight at room temperature and used without further purification. Remarkably, NHS ester dyes can react with primary amines (R-NH_2_) to form an amide bond (Figure S1).

#### 2.1.2. Synthesis of Nano-Arrays

Gold seeds with a diameter of approximately 3 nm, entrapped in negatively charged poly(sodium 4-styrene sulfonate), were prepared according to the standard procedure reported elsewhere for the synthesis of NAs [11].

Briefly, to 20 mL of MilliQ water, 10 µL of poly(sodium 4-styrene sulfonate) (Mw ~70,000 Da, 30 wt.% aqueous solution) and 200 µL of Tetrachloroauric(III) acid solution (25 mM) were added. During vigorous stirring, 200 µL of sodium borohydride (NaBH_4_) (8 mg/mL in water solution) were added, and the solution was further stirred for 2 min.

A 200-µL amount of polyethyleneimine (10 µL/mL) (average Mn ~1800, 50 wt.% aqueous solution), or dye-modified polyethyleneimine, was added to the previously prepared solution and allowed to stir for 30 min at room temperature. The as-synthetized Au nanoparticle arrays were collected by centrifugation (13,400 rpm for 4 min), suspended in 2 mL of MilliQ water, and sonicated for 2 min.

#### 2.1.3. Synthesis of dNAs

In a 100 mL round-bottomed flask, 70 mL of ethanol, 2.4 mL of ammonium hydroxide solution (30 wt.% aqueous solution), 40 µL of tetraethyl orthosilicate (TEOS, 98%), and 2 mL of the as-synthesized Au nanoparticle arrays solution was added, and the resulting solution was allowed to stir for 3 h at room temperature. dNAs and dNAs-Alexa680 were collected by 30 min of centrifugation at 4000 rpm, washed twice with ethanol to remove unreacted precursors and stored in 1 mL of ethanol.

### 2.2. Characterizations of Nano-Architectures

#### 2.2.1. Dynamic Light Scattering (DLS) Measurements

The hydrodynamic diameter and zeta potential measurements were performed using a Malvern Zetasizer Nano ZS90 (Malvern Panalytical Ltd., Malvern, UK).

During measurements, the NPs were resuspended in PBS at pH 7.4 and sonicated for 5 min. The reported values are the average of five consecutive measurements.

#### 2.2.2. UV–Vis Spectrophotometry

The absorption spectra were obtained by means of a double-beam Jasco V-550 spectrometer (Jasco International, Tokyo, Japan).

MilliQ water was employed as a solvent, and the samples were placed in 10 mm path length quartz cuvettes.

#### 2.2.3. Fluorescence Spectrometry

Fluorescence spectra were collected using a Cary Eclipse fluorimeter (Varian Cary Eclipse, Agilent Technologies, Santa Clara, CA, USA) equipped with quartz cuvettes of 1.5 mm path lengths. Excitation and emission slits of 10 nm were employed with a photomultiplier voltage of 700 V. Samples were dispersed in MilliQ water before collecting the spectra.

#### 2.2.4. Fourier Transform–Infrared (FT–IR) Spectroscopy 

Infrared spectra were recorded using a Diamond ATR sampling block for a Cary 630 FTIR (Agilent Technologies, Santa Clara, CA, USA). The spectra of polymers were obtained from around 10 µL of liquid samples (50% in water). The spectra of NAs and dNAs were recorded with around 100 µg of freeze-dried samples. Briefly, the stored solution was centrifuged at 13,400 rpm for 5 min, suspended in 500 µL of MilliQ water, sonicated for 5 min, and freeze-dried overnight.

#### 2.2.5. Electron Microscopy

Transmission electron microscopy (TEM) observation of nanoparticles was carried out on a ZEISS Libra 120 TEM (Carl Zeiss NTS, Oberkochen, Germany), operating at an accelerating voltage of 120 kV and equipped with an in-column omega filter. The colloidal solutions were deposited on 300-mesh carbon-coated copper grids and dried overnight before observation.

#### 2.2.6. Inductively Coupled Plasma–Mass Spectrometry (ICP–MS) Analysis

The gold quantification was performed by dissolving dNAs in 1 mL of aqua regia and digesting them at 200 °C under microwave irradiation with CEM Discover SP-D digestion microwave (CEM, Matthews, NC, USA). The resulting solution was diluted in 5 mL of 3% nitric acid solution, and the Au content was determined by ICP–MS Agilent 7700 (Agilent Technologies, Santa Clara, CA, USA) analysis against a standard calibration curve.

#### 2.2.7. Silica Shell Dissolution Test

For the biodegradation experiments with 20% human serum, Scanning Electron Microscopy (SEM) imaging was performed with a ZEISS Merlin SEM (Zeiss, Oberkochen, Germany), operating at an accelerating voltage of 5 or 10 kV. Briefly, dissolution experiments were conducted as follows: 1 mL of dNAs was precipitated by 5 min of centrifugation at 13,400 rpm and suspended in 800 μL of a PBS (1X) buffer and 200 μL of human serum. The resulting solution was left stirring at 550 rpm at 37 °C. After a certain time (0, 2, 4, 6, 12, and 24 h), a droplet of the colloidal solution was deposited on a silicon wafer and imaged with SEM after oxygen plasma treatment.

### 2.3. Cell Culture

MIA PaCa-2, SCC-25, and UPCI:SCC154 cell lines were purchased from American Type Culture Collection (ATCC, Manassas, VA, USA). SCC-25 (ATCC® CRL-1628™) was maintained in a complete Dulbecco’s modified Eagle medium (DMEM)/F12 medium (1:1), while UPCI:SCC154 (ATCC^®^ CRL-3241™) and MIA PaCa-2 (ATCC^®^ CRM-CRL-1420™) were grown in DMEM from Invitrogen (Carlsbad, CA). Both growth mediums were supplemented with 10% fetal bovine serum (FBS), 4 mM L-glutamine, 1 mM sodium pyruvate, 100 U/mL penicillin, and 100 mg/mL streptomycin (Invitrogen). The SCC-25 medium was also supplemented with 400 ng/mL of hydrocortisone. Cells were maintained at 37 °C in a humidified 5% CO_2_ atmosphere.

#### 2.3.1. Confocal Microscopy

For confocal microscopy experiments, 2.5 × 10^5^ cells were seeded 24 h before experiments in a glass-bottom Petri dish (WillCo-dish GWSt-3522) to reach 70–80% of confluence. Then, cells were treated with dNAs-Alexa680 (max. 2 µg of gold) for 2 h at 37 °C. Right before the end of incubation, Hoechst 33342 and LysoTracker™ Green DND-26 were added to label nuclei and lysosomes, respectively. After incubation, cells were washed twice with PBS and imaged with a Zeiss LSM 880 microscope (Zeiss, Oberkochen, Germany), associated with a thermostated chamber at 37 °C (Zeiss). Cells were imaged with a 40X oil objective using 405, 488, and 647 lasers for nuclei, lysosomes, and dNAs, respectively. At least 5 images with the same magnification were acquired for each cell line and analyzed with Fiji ImageJ software (ImagJ, Wayne Rasband National Institutes of Health). A JACoP plugin was used for colocalization analysis and Pearson’s coefficient.

#### 2.3.2. Cell Viability Assay

For each cell line, 10^4^ cells were seeded in each well of a 96-well plate and, after 24 h, were treated with an increasing amount of dNAs for 2 h at 37 °C. Then, cells were washed twice with PBS, and viability was monitored by a WST-8 assay. Briefly, a WST-8 assay is a viability assay based on the conversion of tetrazolium salt, 2-(2-methoxy-4-nitrophenyl)-3-(4-nitrophenyl)-5-(2,4-disulfophenyl)-2Htetrazolium monosodium salt, to its reduced colored form. For the assay, 10% of a WST-8 reagent was added to the medium, and absorbance (at 450 nm) was measured using a microplate reader (Glomax Discover, Promega, Madison, WI, USA). The percentage of cell viability was determined by comparing drug-treated cells with untreated cells (100% viability). The data represent the average of three independent experiments. Error bars state the standard error from three independent experiments. Two-way ANOVA Dunnett’s multiple comparison analysis was performed on these data.

## 3. Results and Discussion

**Figure 1:**
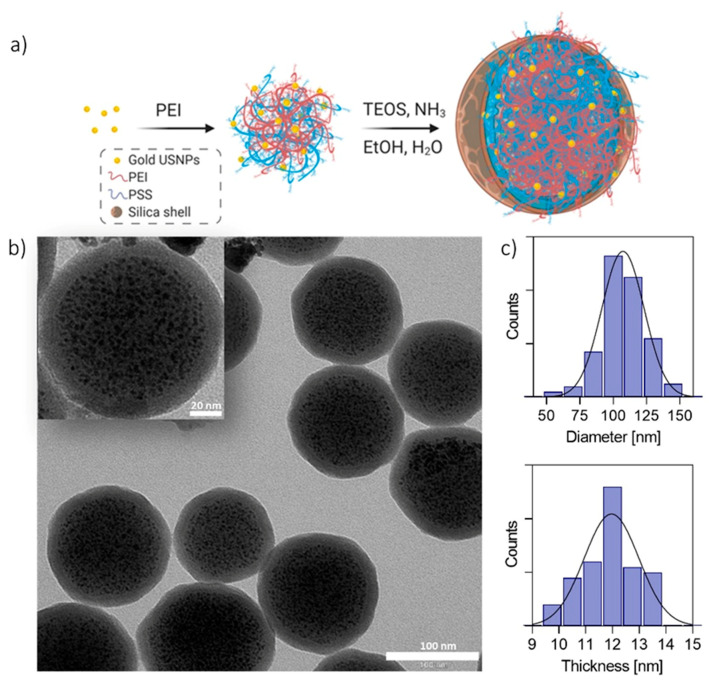
(**a**) Scheme for the synthesis of dragon fruit nano-architectures (dNAs). Gold seeds are synthesized in the presence of poly(sodium 4-styrenesulfonate) (PSS) and assembled in controlled aggregates with polyethyleneimine (PEI). The aggregates are employed as templates for the formation of silica nanocapsules. (**b**) Typical transmission electron microscopy (TEM) image of dNAs. Scale bar: 100 nm. Inset: zoom on one dNA. Scale bar: 20 nm. (**c**) Size diameter distribution and silica shell thickness histograms of dNAs, performed on 150 nanoparticles.

In this work, a new class of ultrasmall-in-nano architectures, dNAs (dragon fruit nanoarchitectures), had been produced by an optimized two-step protocol, as schematically reported in Figure 1a and Figure S1 (†ESI): (i) self-assembly of the controlled aggregates of the polymer (PEI) and ultrasmall gold nanoparticles, followed by (ii) the formation of a hollow silica shell on those templates.

Briefly, controlled aggregates made of poly(sodium 4-styrene sulfonate) (PSS) coated gold ultrasmall nanoparticles (USNPs) of about 2.8 ± 0.4 nm, and the cationic polymer polyethyleneimine (PEI) was formed, exploiting the ionic interaction between the partial charges of the two polymers. A protective, hollow silica nanocapsule was grown on the aggregates, which acted as templates, by following the Stöber method. It is worth remembering that the formation of the hollow silica nanostructures is due to the joint presence of amines from the cationic polymer and aromatic groups of PSS [20]. The presence of PEI in the as-synthesized dNAs was confirmed by IR spectroscopy (Figure S2, †ESI). The results have shown that the spectrum of dNAs mainly differs from the spectrum of NAs for three peaks, which are more intense in PEI, with the peaks appearing first at 1475 cm^−1^ and the other two at around 2840 cm^−1^ and 2942 cm^−1^ (marked in Figure S2. †ESI), all due to C-H vibrations, bending, and stretching [21]. Remarkably, the protocol has demonstrated a significant batch-by-batch reproducibility (Table S1, †ESI), and the composed nano-architectures are stable for almost a year at room temperature (RT) and intrinsically sterile because they are prepared and stored in ethanol. Interestingly, the introduction of PEI has not substantially affected the optical features, nor the external morphology, of the nano-architectures (Figure 1, Table 1, Table S1, Figures S3 and S4, †ESI). Indeed, the resulting nanostructures have a diameter of 107.5 ± 16.6 nm and a shell thickness of 12.0 ± 0.9 nm (Figure 1c). Thus, the size of the dNAs is comparable to standard NAs, while the shell is about half the thickness (Table 1). The formation of a thinner silica shell can be ascribed to the high number of positively charged amino groups of PEI able to support the localized hydrolysis of monomers of TEOS on the surface of the polymeric aggregates [22].

On one hand, it can be useful to synthetically report the standard mechanism of silica nanoparticle formation following the Stöber reaction: (i) hydrolysis of TEOS to orthosilicic acid after its introduction in the reaction medium, (ii) polymerization of orthosilicic acid when the concentration exceeds the saturation limit in ethanol (about 0.02–0.03%), (iii) condensation of low to high molecular weight polymers to 1–2 nm nuclei, and (iv) increase of nuclei size by following a LaMer growth pattern until a 5–7 nm diameter is reached [23,24]. This process continues until the concentration of orthosilicic acid in the reaction medium exceeds the saturation limit. 

The relative amount of gold in dNAs is increased to 9.6 ± 0.8 *w*/*w* (with respect to the 5.9 ± 1.3 w/w of standard NAs), probably due to the thinner silica shell.

The 60% increase of gold loading in dNAs, with respect to the state-of-the-art NAs, is of particular interest for their potential employment as sensitization agents in radiotherapy and radiodiagnostics probes, because the minimum required gold concentration in neoplasms for these techniques is, respectively, 0.1 and 0.5 mg/mL [25,26].

**Figure 2:**
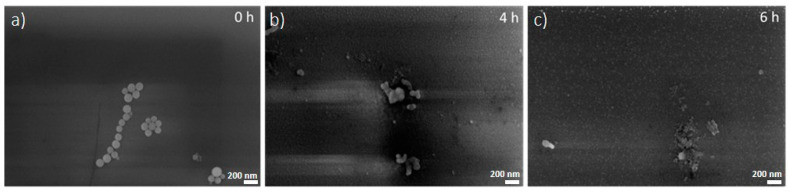
SEM images of dNAs deposited on silicon wafers after (**a**) 0 h, (**b**) 4 h, and (**c**) 6 h of incubation in 20% human serum at 37 °C. Scale bar: 200 nm.

Indeed, the X-ray attenuation properties of nanostructured gold may avoid the limitations of the conventional agents in radio-oncology, among which are toxicity, osmolality, and viscosity [5,25,27]. One of the most important features of dNAs is their faster degradation compared with NAs in biological fluids, another factor probably ascribable to the reduced silica capsule thickness, which takes less time to degrade (Figure 2). It is worth remembering that the (bio)degradation of NAs has already been investigated and results in biosafe building blocks (silicic acid, biodegradable polymers, and glutathione-coated USNPs), which are metabolized or excreted by organisms [3,9,10,14]. The dNA degradation assessment was performed by SEM imaging of samples incubated in 20% human serum at 37 °C (Figure 2). After a few hours of incubation, the nano-architectures started losing their spherical shape, while after 6 h, no more intact dNAs were observed, even if some background silica structures on the silicon wafer could be found. The background material was probably due to the silica re-condensation in the close experimental set-up.

Noticeably, this behavior may be particularly significant for the development of diagnostic agents, or for medical tools that have to perform a fast action.

**Figure 3:**
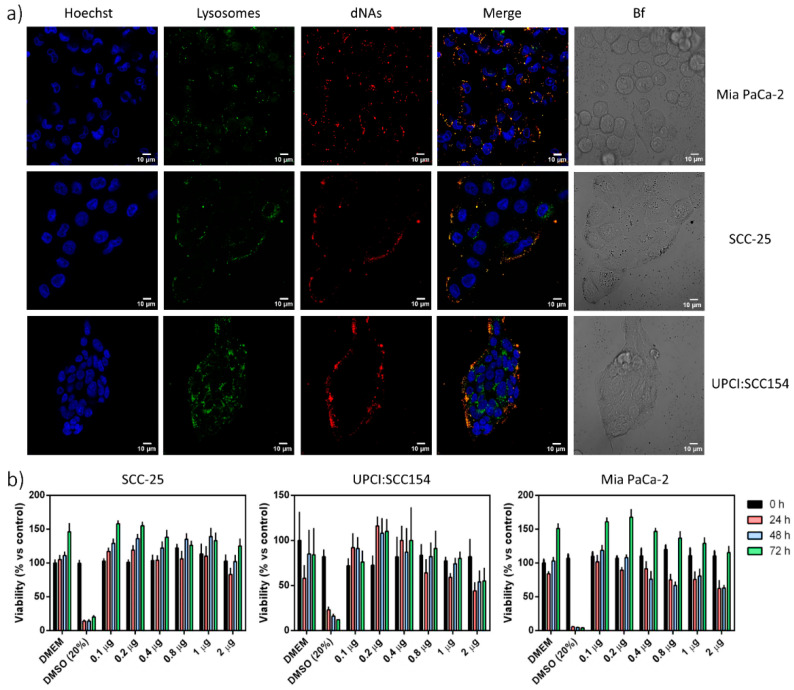
(**a**) Nanoparticle internalization in cells. dNAs-Alexa680 were used to treat different cell lines, and internalization was evaluated by confocal microscopy. From the left to the right column: nuclei (blue, stained with Hoechst 33342), lysosomes (green, stained with Lysotracker green DND-26), dNAs (red, labeled with Alexa-680), superimposition of nuclei, lysosomes, and nanoparticles, and finally the bright field. Scale bar: 10 μm. (**b**) Cytotoxic effect of dNAs on different cell lines. Each cell line was treated with an increasing amount of gold that corresponded to an increasing amount of nanoparticles. Nanoparticles were incubated with cells for 2 h at 37 °C with 5% CO_2_. The viability of each cell line was measured by a WST-8 assay for 72 h after treatment and related to the viability of control cells (treated only with Dulbecco’s modified Eagle medium (DMEM)). Results are the average of three different experiments, and bars state the standard error. (Two-way ANOVA Dunnet’s multiple comparison analysis was performed).

To track dNA internalization behavior and cytotoxicity, three different tumor cell lines were used: pancreatic ductal adenocarcinoma (PDAC) and human papillomavirus (HPV) positive (SCC154) and negative (SCC25) head and neck squamous cell carcinoma (Figure 3a) [4,12,15].

For this purpose, PEI was covalently linked to the commercial AlexaFluor-680 by click chemistry before its use to synthesize the dye-loaded dNAs (Figure S1, †ESI), in agreement with the procedure used for standard NAs [25]. As expected, the absorbance spectrum of dNAs-680 (Figure S5, †ESI) showed the presence of a novel band around 680 nm due to the presence of the dye inside the nanostructure. 

Each cell line was 2D cultured and treated for 2 h with the same amount of dNAs, following other protocols [28,29]. The internalization was qualitatively evaluated by confocal microscopy. A clear fluorescence signal arises from the nano-architectures, confirming efficient internalization by endocytosis in all cell lines, as also confirmed by the high co-localization degree with lysotrackers inside the lysosomes (Figure 3a and Table 2). This result is therefore not surprising, as NAs and dNAs do not show remarkable differences in their dimensions, external surfaces, or comparable zeta potential surface charges. Finally, a WST-8 viability assay was performed on the three cell lines using an increasing amount of gold (corresponding to the increasing amount of dNAs), from 0.1 to 2 µg of gold for each well, in agreement with our standard procedures. The toxicity evaluation was performed for 3 days in order to assess the direct toxicity of both the dNAs and of the building blocks after (bio)degradation.

Even if a transient (but not significant) effect is recorded for the higher doses at the first time point, the dNAs generally demonstrated low toxicity profiles (Figure 3b) in all the cell lines as well as all time points.

It is useful to highlight that all the materials used for both NAs and dNAs are FDA-approved and biocompatible, and both nanoarchitectures share the composition of the shell, which is responsible for biodegradation behavior. Therefore, this process is likely to be similar for both NAs and dNAs. These findings suggest an in vivo biosafety profile similar to standard NAs [3,9,10,14].

## 4. Conclusions

In summary, a cost-effective standardized protocol for the production of degradable ultrasmall-in-nano architectures has been demonstrated, together with significant batch-by-batch reproducibility. Remarkably, the increased gold loading of dNAs may unlock their potential exploitation in radio-oncology and, in particular, for the synergistic co-treatment of some solid neoplasms [30]. Moreover, the presence of PEI in dNAs may also pave the way for their employment in gene delivery and immunotherapy. Our approach, taken together with the next integration with microfluidic systems to promote the semi-automatic scaling up of production, is readily transferable to large-scale manufacturing, paving the way for concrete, cost-effective production of dNAs, representing an important step toward the translation of noble metal nanotheranostics to patients. 

## Figures and Tables

**Table 1:** Comparison between NAs and dNAs.

Nanoparticles Characterizations	NAs	dNAs
DLS Size [nm]	203.1 ± 1.9	222.3 ± 6.3
TEM Size [nm]	107.6 ± 16.1	107.5 ± 16.6
Shell thickness [nm]	18.9 ± 2.2	12.0 ± 0.9
Au loading [w/w%]	5.9 ± 1.3	9.6 ± 0.8
Z-potential [mV]	−20.6 ± 0.4	−20.6 ± 0.6
UV absorbance plasmon [nm]	532	531

DLS—Dynamic light scattering; TEM—Transmission electron microscopy.

**Table 2:** Pearson’s correlation coefficient of the colocalization between dNAs and lysosomes.

Cell Line	Pearson’s Coefficient
SCC-25	0.82 ± 0.08
UPCI:SCC154	0.66 ± 0.12
MIA PaCa-2	0.86 ± 0.04

## Supplementary Materials

The following are available online at https://www.mdpi.com/2079-4991/10/8/1600/s1. Figure S1: Schematic representation of dye-modified dNA synthesis; Figure S2: FT–IR spectra; Table S1: batch-by-batch comparisons; Figure S3: TEM snapshot of NAs and dNAs; Figure S4: UV–Vis spectra; Figure S5: UV–Vis and fluorescence spectra.

## References

[1] Torre L.A., Bray F., Siegel R.L., Ferlay J., Lortet-Tieulent J., Jemal A. (2015). Global cancer statistics, 2012. CA Cancer J. Clin..

[2] Vlamidis Y., Voliani V. (2018). Bringing again noble metal nanoparticles to the forefront of cancer therapy. Front. Bioeng. Biotechnol..

[3] Cassano D., Mapanao A., Summa M., Vlamidis Y., Giannone G., Santi M., Guzzolino E., Pitto L., Poliseno L., Bertorelli R. (2019). Biosafety and biokinetics of noble metals: The impact of their chemical nature. ACS Appl. Bio Mater..

[4] Cassano D., Santi M., D’Autilia F., Mapanao A.K., Luin S., Voliani V. (2019). Photothermal effect by NIR-responsive excretable ultrasmall-in-nano architectures. Mater. Horiz..

[5] Cui L., Her S., Borst G.R., Bristow R.G., Jaffray D.A., Allen C. (2017). Radiosensitization by gold nanoparticles: Will they ever make it to the clinic?. Radiother. Oncol..

[6] Yang X., Yang M., Pang B., Vara M., Xia Y. (2015). Gold nanomaterials at work in biomedicine. Chem. Rev..

[7] Siddique S., Chow J.C.L. (2020). Gold nanoparticles for drug delivery and cancer therapy. Appl. Sci..

[8] Cassano D., Pocoví-Martínez S., Voliani V. (2018). Ultrasmall-in-nano approach: Enabling the translation of metal nanomaterials to clinics. Bioconjugate Chem..

[9] Mapanao A.K., Giannone G., Summa M., Ermini L., Zamborlin A., Santi M., Cassano D., Bertorelli R., Voliani V. (2020). Biokinetics and clearance of inhaled gold ultrasmall-in-nano architectures. Nanoscale Adv..

[10] Cassano D., Summa M., Pocoví-Martínez S., Mapanao A.K., Catelani T., Bertorelli R., Voliani V. (2018). Biodegradable ultrasmall-in-nano gold architectures: Mid-period in vivo distribution and excretion assessment. Part. Part. Syst. Charact..

[11] Cassano D., Rota Martir D., Signore G., Piazza V., Voliani V. (2015). Biodegradable hollow silica nanospheres containing gold nanoparticle arrays. Chem. Commun..

[12] Mapanao A.K., Santi M., Faraci P., Cappello V., Cassano D., Voliani V. (2018). Endogenously triggerable ultrasmall-in-nano architectures: Targeting assessment on 3D pancreatic carcinoma spheroids. ACS Omega.

[13] Armanetti P., Pocoví-Martínez S., Flori A., Avigo C., Cassano D., Menichetti L., Voliani V. (2018). Dual photoacoustic/ultrasound multi-parametric imaging from passion fruit-like nano-architectures. Nanomedicine.

[14] d’Amora M., Cassano D., Pocoví-Martínez S., Giordani S., Voliani V. (2018). Biodistribution and biocompatibility of passion fruit-like nano-architectures in zebrafish. Nanotoxicology.

[15] Mapanao A.K., Voliani V. (2020). Three-dimensional tumor models: Promoting breakthroughs in nanotheranostics translational research. Appl. Mater. Today.

[16] Shi J., Kantoff P.W., Wooster R., Farokhzad O.C. (2017). Cancer nanomedicine: Progress, challenges and opportunities. Nat. Rev. Cancer.

[17] Vicennati P., Giuliano A., Ortaggi G., Masotti A. (2008). Polyethylenimine in medicinal chemistry. Curr. Med. Chem..

[18] Benjaminsen R.V., Mattebjerg M.A., Henriksen J.R., Moghimi S.M., Andresen T.L. (2013). The Possible “proton sponge” effect of polyethylenimine (PEI) does not include change in lysosomal pH. Mol. Ther..

[19] Pocoví-Martínez S., Cassano D., Voliani V. (2018). Naked nanoparticles in Silica Nanocapsules: A versatile family of nanorattle catalysts. ACS Appl. Nano Mater..

[20] van Bommel K.J.C., Jung J.H., Shinkai S. (2001). Poly(L-lysine) aggregates as templates for the formation of hollow silica spheres. Adv. Mater..

[21] Yudovin-Farber I., Beyth N., Weiss E.I., Domb A.J. (2010). Antibacterial effect of composite resins containing quaternary ammonium polyethyleneimine nanoparticles. J. Nanopart. Res..

[22] Kind L., Shkilnyy A., Schlaad H., Meier W., Taubert A. (2010). Poly(ethylene oxide)-poly(ethylene imine) block copolymers as templates and catalysts for the in situ formation of monodisperse silica nanospheres. Colloid Polym. Sci..

[23] Masalov V.M., Sukhinina N.S., Kudrenko E.A., Emelchenko G.A. (2011). Mechanism of formation and nanostructure of Stöber silica particles. Nanotechnology.

[24] Stöber W., Fink A., Bohn E. (1968). Controlled growth of monodisperse silica spheres in the micron size range. J. Colloid Interface Sci..

[25] Lee N., Choi S.H., Hyeon T. (2013). Nano-sized CT contrast agents. Adv. Mater..

[26] Al Zaki A., Joh D., Cheng Z., De Barros A.L.B., Kao G., Dorsey J., Tsourkas A. (2014). Gold-Loaded polymeric micelles for computed tomography-guided radiation therapy treatment and radiosensitization. ACS Nano.

[27] Kunz-Schughart L.A., Dubrovska A., Peitzsch C., Ewe A., Aigner A., Schellenburg S., Muders M.H., Hampel S., Cirillo G., Iemma F. (2017). Nanoparticles for radiooncology: Mission, vision, challenges. Biomaterials.

[28] Cassano D., Santi M., Cappello V., Luin S., Signore G., Voliani V. (2016). Biodegradable passion fruit-like nano-architectures as carriers for cisplatin prodrug. Part. Part. Syst. Charact..

[29] Santi M., Maccari G., Mereghetti P., Voliani V., Rocchiccioli S., Ucciferri N., Luin S., Signore G. (2017). Rational design of a transferrin-binding peptide sequence tailored to targeted nanoparticle internalization. Bioconjugate Chem..

[30] Santi M., Mapanao A.K., Cassano D., Vlamidis Y., Cappello V., Voliani V. (2020). Endogenously-activated ultrasmall-in-nano therapeutics: Assessment on 3D head and neck squamous cell carcinomas. Cancers.

