# Supplementary material for: A Cost-Effective Approach for Non-Persistent Gold Nano-Architectures Production
Source: Nanomaterials (Basel). 2020 Aug 14;10(8):1600. doi: 10.3390/nano10081600 (PMC7466706; doi:10.3390/nano10081600)
Supplement: Supplementary file 1 [file nanomaterials-10-01600-s001.pdf]

nanomaterials

Supplementary Materials: A Cost-Effective Approach for Non-persistent Gold Nano-Architectures Production

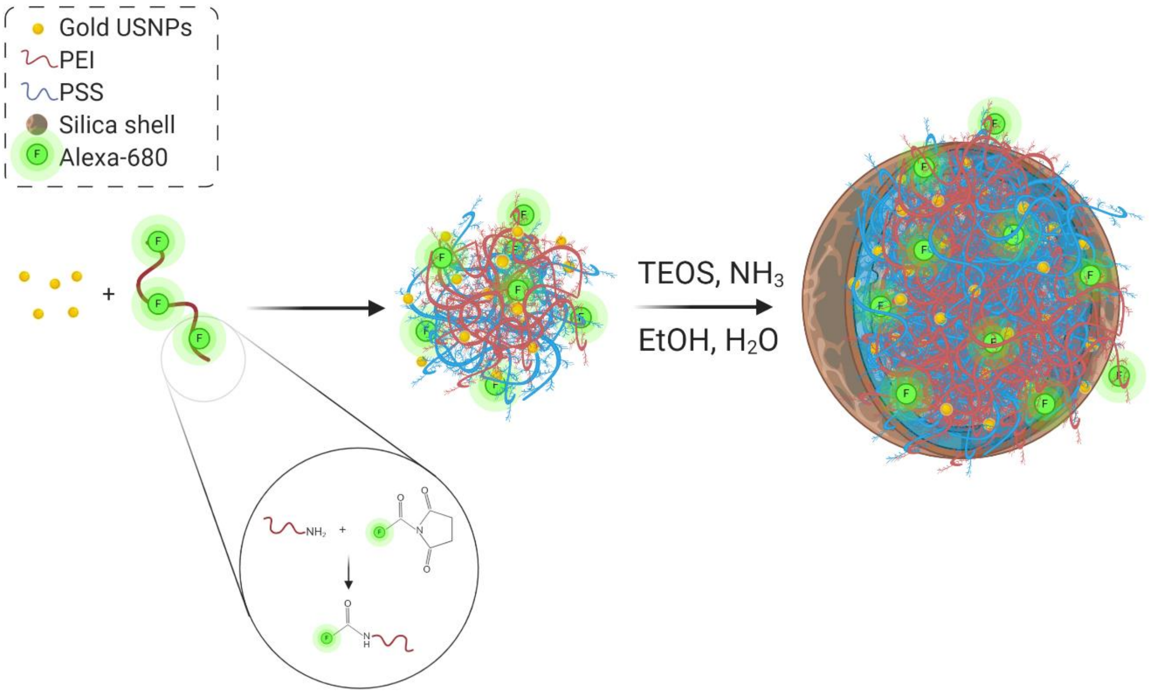

Figure S1. Scheme for the synthesis dye-modified dragon fruit nanoarchitectures (dNAs). Gold seeds are synthesized in the presence of poly(sodium 4-styrenesulfonate) (PSS) and assembled in controlled aggregates with previously functionalized polymer polyethyleneimine (PEI). The aggregates are employed as templates for the formation of silica nanocapsules. Insight of the reaction: the N-Hydroxysuccinimide (NHS) group reacts with the primary amines for the formation of an amide bond.

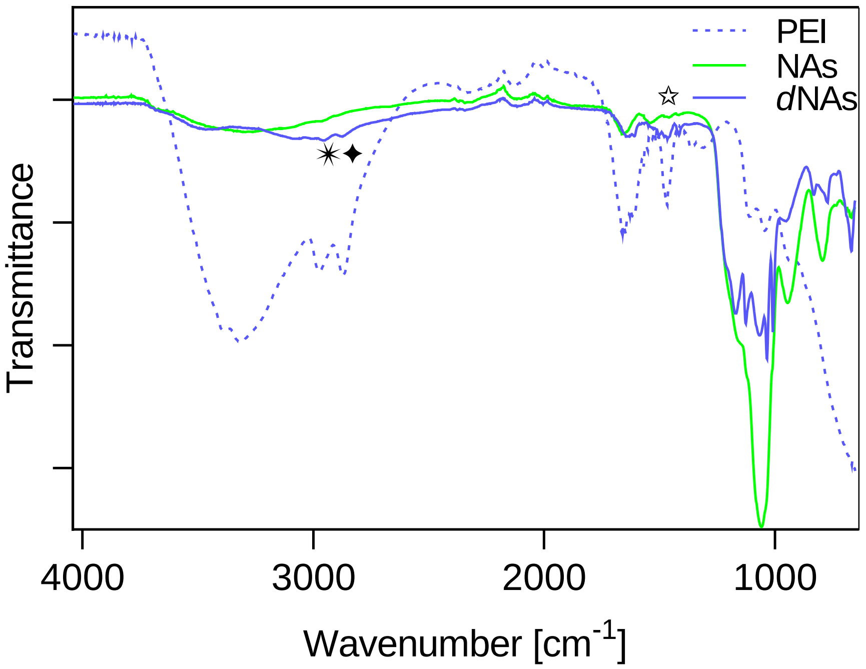

Figure S2. Fourier Transform–Infrared (FT–IR) spectra of passion fruit-like nano-architectures (NAs, containing poly(L-lysine) (PL), green line) and dragon fruit nano-architectures (dNAs, containing PEI, blue line). The blue dotted line is the infrared spectra of PEI. Relevant peaks are marked (✳ 2942 cm^{-1}, ✦ 2840 cm^{-1}, and ☆ 1475 cm^{-1}).

Table S1. Batch-by-batch comparison between three independent dNA syntheses.

Nanoparticles Characterizations	S1	S2	S3
DLS Size [nm]	220.7 ± 7.4	219.3 ± 8.7	226.8 ± 2.8
TEM Size [nm]	107.4 ± 14.5	104.9 ± 19.9	109.3 ± 15.3
Shell thickness [nm]	11.9 ±0.8	11.8 ± 0.9	12.4 ±1.0
Au loading [w/w%]	9.7	8.8	10.4
Z-potential [mV]	−20.6 ± 0.4	−24.4 ± 0.5	−16.9 ± 0.9

DLS—Dynamic light scattering; TEM—Transmission electron microscopy.

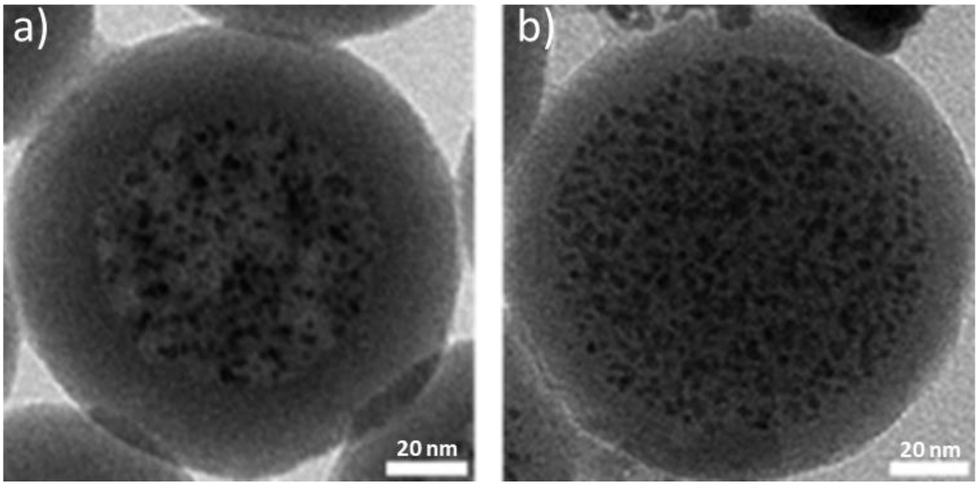

Figure S3. Transmission electron microscopy (TEM) images of NAs (**a**) and dNAs (**b**). Scale bar: 20 nm.

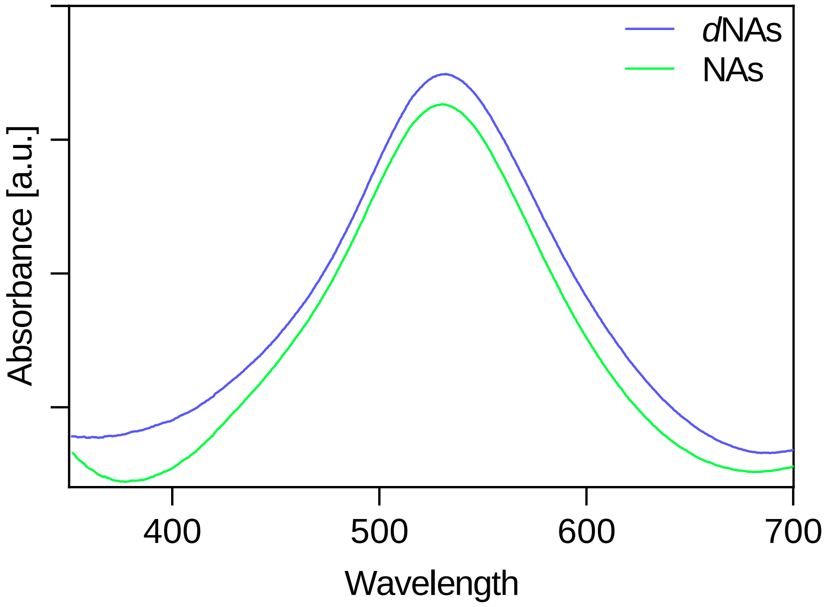

Figure S4. Normalized and background-subtracted UV–vis spectra of dNAs and NAs in MilliQ water.

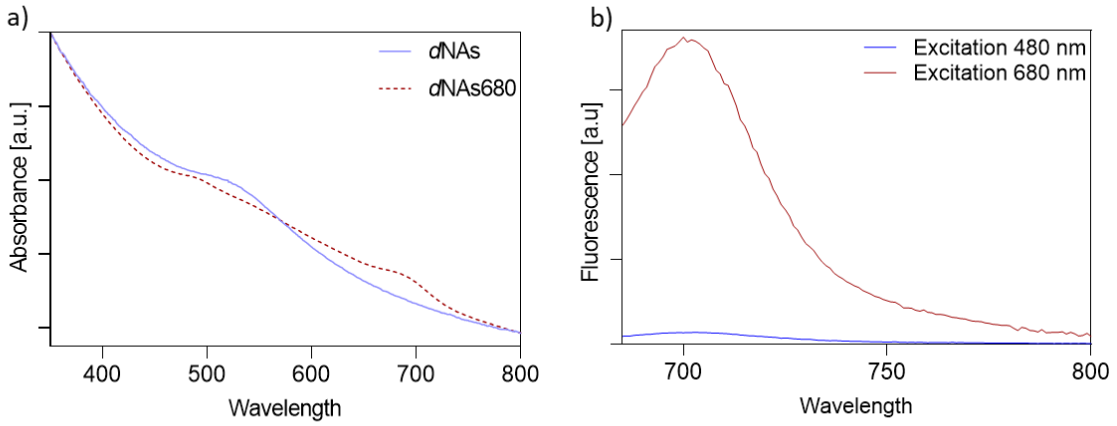

Figure S5. (**a**) Normalized and background-subtracted UV–vis spectra in MilliQ water of dNAs and dye-modified dNAs (continuous red) and (**b**) dNA fluorescence emission spectra in MilliQ water. The red line corresponds to 680 nm excitation and the blue emission line to 480 nm excitation.
